# Early Primary Biliary Cirrhosis: A New Association with Erythema Nodosum of Unknown Origin

**DOI:** 10.1155/2010/121620

**Published:** 2010-07-25

**Authors:** Nikolaos K. Gatselis, Kalliopi Zachou, George N. Dalekos

**Affiliations:** ^1^Department of Medicine, School of Medicine, University of Thessaly, Thessaly, 41110 Larissa, Greece; ^2^Research Group of Investigational Medicine, Institute of Biomedical Research and Technology, Centre for Research and Technology-Thessaly (CE.RE.TE.TH), 41222 Thessaly, Larissa, Greece

## Abstract

Primary biliary cirrhosis (PBC) is associated with immune-mediated dermatologic disorders. The association of PBC with erythema nodosum (EN) seems rare. We report two females (42 and 44 years old) with low-grade fever, arthralgias, and elevated cholestatic enzymes in the first and fatigue in the second. Patients were also suffering from typical EN lesions characterized by multiple erythematous, painful nodules over the anterior portions of their lower extremities. Clinical and extensive laboratory work up excluded all known EN causes. PBC diagnosis was established according to the cholestatic biochemical profile, anti-mitochondrial antibodies (AMA) positivity and liver histology (first), and AMA and antinuclear (ANA) PBC-specific antibodies (second). Our report may suggest that PBC could be kept in mind in EN patients of unknown aetiology and particularly, when middle-aged female patients are affected. In such cases a thorough evaluation for AMA and/or ANA PBC-specific antibodies could be helpful to achieve a correct and timely diagnosis.

## 1. Introduction

Erythema nodosum (EN) is the most common form of paniculitis. It is a cutaneous reaction pattern characterized clinically by the presence of erythematous tender nodules and raised plaques. They tend to be symmetrical in distribution and are usually located bilaterally on the lower extremities, particularly on the anterior tibial surface, although they may also involve the ankles, the lower parts of the thighs, and the forearms [1–4]. Although EN usually has no specific documented cause, it is imperative to investigate possible triggers. Among them, streptococcal infections, tuberculosis, inflammatory bowel disease (IBD), drug reactions, infective endocarditis, and sarcoidosis are the most common causes in children and adults [1–4].

Primary biliary cirrhosis (PBC) is an autoimmune cholestatic liver disease, which affects mainly middle-aged women and is characterized by a progressive immune mediated inflammatory destruction of the small intrahepatic bile ducts with portal inflammation leading to cirrhosis and subsequent liver failure [5, 6]. The diagnostic hallmark of the disease is the detection of antibodies reactive to mitochondria antigens (AMA) [7–9], though several other autoantibodies have been detected either disease-specific or not [7, 10–13]. The disease is frequently associated with a variety of extrahepatic autoimmune or immune-mediated conditions, including Sjogren's syndrome, Hashimoto's thyroiditis, scleroderma, Henoch-Schonlein purpura, and antiphospholipid syndrome [6, 14–16]. However, so far the association of PBC with EN seems to be extremely rare. Indeed, to the best of our knowledge, only one case of PBC associated with EN has been reported in the English literature [17]. Herein, we report two cases of early PBC associated with EN and discuss the potential relationship among these conditions. Both patients gave oral and written consent for the study.

## 2. Case Reports

### 2.1. Patient 1

A 42-year-old woman was admitted because of low-grade fever, arthralgias and painful red nodules on her legs during the last ten days. Her family and past history were unrevealing. Physical examination revealed palpable liver and multiple bilateral tender erythematous nodules on her lower extremities typical of EN. Abnormal laboratory tests were as follows: haemoglobin 10.5 g/dL, platelets 665000/*μ*L, erythrocyte sedimentation rate (ESR 99 mm/h), aspartate aminotransferase (AST) 45 U/L (upper normal limit, UNL: 40 U/L), alanine aminotransferase (ALT) 83 U/L (UNL: 40 U/L), gamma-glutamyl transpeptidase (*γ*-GT) 515 U/L (UNL: 37 U/L) and alkaline phosphatase (ALP) 480 U/L (UNL: 124 U/L), and serum immunoglobulin M (IgM) levels 385 mg/dL (UNL: 200 mg/dL). Ultrasound of the upper abdomen, chest X-ray, and ECG were normal. Clinical and extensive laboratory examinations using conventional and molecular techniques [18] ruled out known causes of EN like IBD, sarcoidosis, and several infectious diseases including streptococcal infections, hepatitis B, hepatitis C and human immunodeficiency virus, herpes simplex virus, cytomegalovirus, adenovirus, Echo and Coxsackie viruses, Epstein-bar virus and brucellosis, leptospirosis, or tuberculosis.

However, liver autoimmune serology tests revealed positive results for antinuclear antibodies by indirect immunofluorescence (IIF) on HEp2 cells (ANA: 1/160; positive titre >1/40) with multiple nuclear dot (MND) pattern and AMA: 1/160 by IIF on in-house rat multiorgan substrate panel that included kidney, liver, and stomach using standard protocols (positive titre >1/20) [7, 11, 19, 20]. The AMA were also detected by an enhanced performance M2 IgG-isotype-specific ELISA (45 Units; UNL: 20 Units) according to the manufacturer's instructions (ELISA; Quanta Lite, INOVA Diagnostics, San Diego, CA) and blotting [7–9]. A complete investigation for other organ- and non-organ-specific autoantibodies as anti-thyroid antibodies, anti-dsDNA, anti-Ro, anti-La, anti-Sm, anti-sp100, and anti-gp210 autoantibodies was unrevealing. A liver biopsy was consistent with stage I of PBC according to Ludwig's classification. A diagnosis of early PBC associated with EN was then made based on the cholestatic biochemical profile, the detection of AMA, elevated IgM levels and the liver histology. The patient was treated for both PBC and EN with prednisolone (15 mg/day with gradual tapering) and ursodeoxycholic acid (13.5 mg/kg/day). She gradually responded and after 3 months she had normal liver function tests (LFTs), she was out of corticosteroids use, and had no symptoms. During the last follow-up she was still in good health with normal LFTs and free of symptoms (only under ursodeoxycholic acid).

### 2.2. Patient 2

A 44-year-old woman was admitted due to fatigue and a history of multiple erythematous, tender nodules over the anterior portions of her lower extremities of 2 years duration characterized by spontaneous remissions and exacerbations. Her family and past medical history were unremarkable. Physical examination revealed only the painful cutaneous lesions in the lower extremities typical of EN. Laboratory data showed only a slight elevation of ESR (33 mm/h). As in *case 1*, after the exclusion of common causes of EN the patient was investigated for autoimmune serology markers. She was proved positive by IIF for ANA of MND pattern (titre: 1/80) and AMA (1/160). Further testing for ANA-PBC-specific antibodies and AMA was done by molecularly based assays, namely, IgG-specific ELISAs (INOVA Diagnostics, San Diego, CA), which proved also positive for anti-sp100 (55.3 Units; UNL: 20 Units) and M2 IgG-isotype specific AMA (92 Units), respectively.

Due to the absence of elevated LFTs liver biopsy considered anethical and a diagnosis of subclinical PBC was made based on the sex and the middle age of the patient along with the stable positive AMA and ANA-PBC-specific antibodies. Treatment with low dose of nonsteroidal anti-inflammatory drugs was initiated with gradual improvement. Of note, there was not any new relapse episode during the follow-up (so far 30 months) though ANA-PBC-specific (anti-sp100) antibodies and AMA have remained high positive with fluctuating titres during the same period. Indeed, four investigations of 6-month intervals during the follow-up period showed the following positive titres of anti-sp100 (ANA-PBC-specific antibodies; 37, 21, 58 and 69 Units) and anti-M2 IgG antibodies (AMA; 80, 65.6, 98.5 and 89.4 Units), although the cholestatic enzymes were within normal limits.

## 3. Discussion

We report here two patients presented with typical features of EN and early PBC. To the best of our knowledge, there has been only one publication in the English literature describing this association [17]. PBC is an autoimmune liver disease characterized by the occurrence of AMA (almost pathognomonic laboratory diagnostic marker) and an association with several other autoimmune or immune-mediated conditions [6, 14–16, 21]. Its diagnosis is often based on a combination of characteristic symptoms including pruritus and profound fatigue though asymptomatic cases at the time of diagnosis are not infrequent, cholestatic liver enzyme disturbances, positive titre of AMA, and characteristic liver biopsy [6, 21].

EN is a common and almost challenging condition in clinical practice. It affects frequently young female patients and the most direct and indirect evidence support the involvement of a type IV delayed hypersensitivity response to numerous and different antigens for its pathogenesis. As a result, EN has aetiologically been associated with an extensive and heterogeneous group of diseases depending on the population characteristics and the geographic location [1]. For instance, streptococcal infections are the most common identifiable aetiology, especially in children, while tuberculosis has been found to be the most common association in areas where the disease is endemic. Drug and hormonal reactions, IBD, and granulomatous diseases, such as sarcoidosis, tuberculosis, and granulomatous colitis have also been reported in common among adults. As actually from the pathophysiological perspective, PBC belongs to the granulomatous diseases, its potential association with EN appears rationale and attractive. Nevertheless, even after an extensive investigation no underlying cause is identified in almost 60% of EN cases [1].

A number of skin conditions have been associated with PBC. A recent case-control study of 49 patients with PBC revealed that 88% of patients also had dermatologic symptoms and, in nearly 40% of these cases, the dermatologic symptom led to the eventual diagnosis of PBC [22]. This seems to be also the case in our patients, where EN was the first major symptom of their underlying early PBC.

In the two cases described in this paper, the diagnosis of PBC is supported by the presence of AMA- and PBC-specific ANA [7, 8, 11, 12]. Liver biopsy was performed in one of them and liver histology was consistent with early PBC (stage I). Clinical and extensive laboratory work-up excluded sarcoidosis and other causes of EN. Of note, LFTs were within normal limits in one patient and therefore, she denied a liver biopsy. However, the stable presence of AMA and ANA-PBC-specific antibodies during follow-up rather excluded false positive serology during the acute setting of EN. In addition, it is well known from the literature that an isolated AMA positive test is indicative of early PBC and that silent disease eventually becomes clinically and biochemically evident up to 18 years after the first AMA screening [23, 24]. Our report demonstrated that early PBC can be the correct diagnosis in female patients presenting with EN after the exclusion of other common causes. The latter could be the case even when LFTs are within normal limits.

Conclusively, since most clinicians do not consider PBC in the differential diagnosis of EN, our report indicates that from the clinical point of view, PBC could be considered in patients with EN of unknown aetiology and particularly, when female patients of middle age are affected irrespective of the presence or absence of elevated cholestatic enzymes. In such cases a thorough evaluation for the detection of AMA- and/or ANA PBC-specific antibodies could be helpful to achieve a correct and timely diagnosis. On the other hand, it would be interesting in the future to determine the prevalence of this association in large cohorts of PBC patients and assess whether the prognosis and outcome of the disease in these cases are different compared to those PBC patients without associated EN.
